# Myasthenic Crisis in an Elderly Patient with Positive Antibodies against Acetylcholine and Anti-MuSK, Successfully Treated with Noninvasive Mechanical Ventilation

**DOI:** 10.1155/2015/624718

**Published:** 2015-12-10

**Authors:** José A. Fernández, Antonio Fernández-Valiñas, Daniel Hernández, Joel Orozco, Antonio Lugo

**Affiliations:** Hospital Ángeles Clínica Londres, Durango No. 50, Roma Norte, Cuauhtémoc, 06700 Ciudad de México, DF, Mexico

## Abstract

Myasthenia gravis is an autoimmune disease characterized by muscle weakness. Subjects with antibodies against acetylcholine usually have greater ocular symptoms, lower bulbar weakness, and fewer respiratory complications, compared to individuals with anti-MuSK antibodies. The presence of positivity to both types of antibodies in the same patient is uncommon, and the clinical behavior of these individuals is uncertain. A myasthenic crisis is characterized by respiratory and bulbar muscle weakness, causing acute respiratory failure which requires mechanical ventilatory support. We present the case of a 73-year-old man with a medical history of myasthenia gravis and positive antibody titers against acetylcholine and anti-MuSK, who sought for medical assessment because of respiratory tract infection symptoms, dysphagia, and generalized weakness. Initially, no respiratory distress was found. After 24 hours the patient showed respiratory deterioration and neurological impairment. Endotracheal intubation was rejected, so ventilatory support with noninvasive ventilation was started. The patient was supported by intense respiratory therapy, and infusion of immunoglobulin was initiated. The individual responded favorably, improving his general condition. Weaning from noninvasive mechanical ventilation was possible after six days. Our case illustrates that noninvasive ventilation, properly supported by intense respiratory therapy, can be a great option to avoid intubation in the myasthenic patient.

## 1. Introduction

Myasthenia gravis is an autoimmune disease characterized by the formation of autoantibodies against nicotinic acetylcholine receptors at the neuromuscular junction or by the presence of antibodies directed against other postsynaptic muscle fiber components like muscle specific tyrosine kinase (MuSK). Clinically it is manifested as muscle weakness of varying intensity [1]. It is recognized that the type of present antibodies determines the clinical behavior of the disease. Antibodies against acetylcholine can be found in young or elderly patients. These subjects usually have more weakness of the limbs in comparison to the bulbar muscles and marked ptosis, and thymoma or thymic hyperplasia is present in 80% of cases. Recurrent crisis is not usual in these patients. Individuals with anti-MuSK antibodies have a more complex clinical course. This group of patients are usually female and under 40 years. They show predominance of the bulbar symptoms, less ocular disturbances, and often lack of thymoma. In addition, they may show poor response to conventional therapy and recurrent myasthenic crisis. The presence of both kinds of antibodies in an individual is very rare, and therefore, the clinical behavior of these patients is extremely uncertain [2].

Myasthenic crisis should be considered a neurological emergency. It is characterized by weakness of the bulbar and respiratory muscles, severe enough to compromise ventilation and airway permeability, causing acute respiratory failure which requires mechanical ventilatory support [3]. Currently, the prognosis of these patients has improved dramatically thanks to the immunomodulatory therapy with plasmapheresis, immunoadsorption, or immunoglobulin. The development and appropriate use of noninvasive ventilation have helped to avoid the complications involved in endotracheal intubation, which has further improved the prognosis of these patients. With proper treatment, mortality from this condition is less than 5% [4].

We report the case of an elderly patient with positive antibodies against acetylcholine and anti-MuSK, who, despite generalized muscular weakness and bulbar muscles involvement, showed an adequate response to noninvasive ventilation.

## 2. Case Presentation

The patient is a 73-year-old male with medical history of diabetes mellitus, hypertension, chronic obstructive pulmonary disease, and obesity grade 2, plus myasthenia gravis of two years of diagnosis, with positive antibody titers against acetylcholine (normal value by immunoradiometric assay <0.25 nmol/L; patient value 6.5 nmol/L) and anti-MuSK (normal value by enzymatic immunoassay <0.4 U/mL; patient value 4.2 U/mL), treated with pyridostigmine (60 mg every 4 hours), azathioprine (50 mg twice a day), and prednisone (5 mg once a day). He is not bearer of thymoma. He had a myasthenic crisis a year ago, which required invasive mechanical ventilation and admission to the intensive care unit for a week. After this event, with established medical treatment, he was wearing an acceptable quality of life.

The individual began his current condition three days before his hospitalization with productive cough, pleuritic pain, and fever. Later he presented difficulty swallowing, nasal voice, ptosis, and generalized weakness. After first medical evaluation in our hospital unit, bulbar symptoms reported by the patient were corroborated along with the weakness of upper and lower extremities, with no observed data of respiratory distress. Clinically, a pulmonary condensation syndrome was integrated in the right hemithorax, so both diagnostics of exacerbation of myasthenia gravis in stage IIa of the Osserman and Genkins classification, and community-acquired pneumonia, were established. The patient entry arterial gases showed no disturbances.

During the first 24 hours of hospitalization, the subject presented respiratory deterioration, referring to dyspnea and showing bradypnea, thoracoabdominal dissociation, and employment of auxiliary breathing muscles. An aggravation in the Osserman and Genkins classification was declared, setting the patient status in stage IVb. Neurologically he was observed to be stuporous, with a Glasgow coma scale of 10 points. Blood gases demonstrated hypoxemia and hypercapnia. Patient was assessed by the critical care service, who indicated need for admission to their unit to start invasive mechanical ventilation and immunomodulatory therapy; however, the responsible relative of the individual did not accept advanced management of the airway and signed a nonintubation order. Therefore keeping the patient in the intermediate care unit and initiating treatment with immunoglobulin (2 grams per kilo of body weight, for 4 days) and noninvasive mechanical ventilation in bilevel continuous positive airway pressure mode, with initial parameters of positive inspiratory airway pressure of 12, positive expiratory airway pressure of 5, inspired oxygen fraction of 60%, and respiratory rate of 20 breaths per minute, were decided. A CareFusion* VELA* ventilator in noninvasive mechanical ventilation mode was employed. Blood gases prior to the start of noninvasive mechanical ventilation showed pH of 7.34, pO_2_ of 54.1 mmHg, pCO_2_ of 44 mmHg (normal values for Mexico City 28–32 mmHg), and HCO_3_ of 28.3 mmol/L (normal values for Mexico City 18 to 22 mmol/L). Initially the use a nasal mask was decided, but the patient was not able to keep his mouth closed due facial muscle weakness, which generated a big leak of air. For this reason, changing to an orofacial mask was necessary. After one hour of noninvasive respiratory therapy, the patient showed gasometric improvement with a pH of 7.36, pO_2_ of 64 mmHg, pCO_2_ of 36.4 mmHg, and HCO_3_ of 29.1 mmol/L. He also presented significant neurological amelioration, recovering alertness, but at times he showed restlessness and uncooperativeness, so an infusion of dexmedetomidine was started at a dose of 0.5 *μ*g/kg/hr. With this measure a better coupling to the noninvasive ventilatory therapy was achieved. Clinically the patient kept an adequate respiratory frequency, with no use of the accessory muscles of respiration and no thoracoabdominal dissociation. Due to the significant bulbar weakness presented by the individual, the face mask was recurrently removed to allow coughing, suctioning of secretions, and pulmonary percussion therapy. Micronebulization treatment with ipratropium bromide was initiated to prevent bronchospasm. After six hours, the patient showed a complete recovery of neurological status. Dexmedetomidine infusion was no longer necessary. The gas analysis reflected total remission of hypoxemia and hypercapnia, with pH of 7.45, pO_2_ of 84 mmHg, pCO_2_ of 28 mmHg, and HCO_3_ of 24.3 mmol/L. Oxygen saturation was 97%.

After 48 hours of continuous therapy with noninvasive ventilation and immunoglobulin treatment, the patient was able to restart the oral route. Besides removing of noninvasive mechanical ventilation mask to allow the elimination of secretions, patient was put on pap diet with assisted technique and was allowed to rest for the ventilatory support during two hours after every meal. During resting times, the patient was maintained with a venturi mask with an inspired oxygen fraction of 60%. Blood gases kept in acid-base balance and hypoxemia did not recur.

As part of the support management, starting prophylactic anticoagulation with low molecular weight heparin, protection of the gastric mucosa with proton pump inhibitor, and glycemic control by insulin scheme every six hours and basal insulin was decided. Hemodynamic monitoring with telemetry was established. Treating the respiratory tract infection with third-generation cephalosporin and clarithromycin was decided. The treatment with azathioprine was maintained and pyridostigmine was put off until the patient was able to completely retake the oral route. The chronic steroid treatment was kept intravenously until the patient was able to take it orally.

After completing four days of immunoglobulin therapy and noninvasive mechanical ventilation, the patient evolved favorably, leaving the bilevel positive airway pressure therapy during the day and keeping it only overnight. Patient status was set in stage IIa of Osserman and Genkins. Solid diet was initiated and adequately tolerated, and significant improvement in lung infection that triggered the myasthenic crisis was observed. On the sixth day, pulmonary rehabilitation was initiated with incentive spirometry, and noninvasive mechanical ventilation was withdrawn definitely. After seven days of treatment, the patient was discharged home with his usual treatment.

## 3. Discussion

Respiratory infections are one of the most common etiologies in the development of myasthenic crisis. Our patient, after starting with clinical data from respiratory infection three days before seeking medical attention, suddenly evolved from class IIa of the Osserman and Genkins score to class IVb, demonstrating how quickly the respiratory impairment in these diseases can be presented, demanding mechanical ventilatory support at any time.

The vast majority of patients with respiratory failure of neuromuscular origin initially develop CO_2_ retention, and, particularly in patients with myasthenic crisis, hypoxemia is often seen late [4]. Our patient, however, quickly showed a mixed respiratory failure, probably triggered both by weakness of the respiratory muscles and by alveolar commitment caused by infection. Because of the clinical data of respiratory failure, bulbar weakness, gasometric evidence of hypoxemia and hypercapnia, and neurological deterioration observed in the patient, endotracheal intubation and the use of invasive mechanical ventilation was initially decided. Noninvasive mechanical ventilation appeared as a therapeutic resource of great value after the nonintubation order signed by the relative of the patient.

Because the noninvasive ventilatory support in continuous positive airway pressure mode (cPAP) does not actively assist during inspiration [5], the myasthenic patient usually responds more favorably to the bilevel positive airway pressure mode (BiPAP) that delivers adjustable degrees of continuous positive airway pressure, which is higher during inspiration and lower during expiration. Each cycle begins with the breathing efforts of the patient. The positive inspiratory airway pressure helps to decrease the resistance of the upper airway, reducing the work of breathing. The positive end-expiratory airway pressure prevents airway collapse before the end of the respiratory cycle, reducing the risk of atelectasis. This type of ventilatory support is particularly effective in patients with neuromuscular weakness, who are capable of starting the respiratory cycle but are unable to mobilize sufficient amounts of air to prevent the development of atelectasis [6].

The noninvasive ventilation is strongly contraindicated in patients who cannot protect their airway and in subjects who are unable to effectively manage secretions despite receiving support by aspiration and respiratory therapy [5]. In myasthenia gravis, the presence of anti-MuSK antibodies is associated with marked bulbar symptoms, which tend to cause problems in swallowing and difficulty in handling secretions. In the patient in myasthenic crisis, the exacerbation of these symptoms is an inconvenience for the use of noninvasive mechanical ventilation [3]. Although there are various face masks, and most guidelines of noninvasive ventilatory support do not give superiority to any in particular [7], aspiration and secretions management is facilitated by using a nasal mask; however, this has the disadvantage of generating a large air leak if the patient keeps his mouth open. Our patient, due to muscle weakness, was unable to keep his mouth closed. For this reason it was preferred to place an orofacial mask, withdrawing it frequently for aspiration and to allow coughing. Despite this measure, noninvasive ventilation might have failed if it was not for the excellent response after the initiation of the treatment with immunoglobulin and the intense respiratory therapy given. In addition to continuous suctioning, the patient received lung physiotherapy very often, his position was changed up to six times per day, and he was treated with bronchodilators to prevent bronchospasm. These measures, often neglected, prevent pulmonary complications in patients with myasthenic crisis [8]. After starting the pap diet, delaying the placement of BiPAP up to two hours after meals was decided to avoid episodes of aspiration.

Some predictors of response to noninvasive mechanical ventilation in the myasthenic patient have been identified. Seneviratne et al. conducted a retrospective cohort study of 60 episodes of myasthenic crisis. They found that pCO_2_ greater than 45 mmHg prior to initiation of treatment with BiPAP is a failure predictor in the use of this therapy. In this same study, the initiation of noninvasive mechanical ventilation prevented intubation in 14 of 24 patients, and the only variable that showed decrease in the duration of endotracheal intubation was the early use of BiPAP [6]. Another retrospective study by Wu et al. showed that an APACHE II score of less than 6 points and a serum bicarbonate below 30 mmol/L are predictors of BiPAP therapy success in myasthenic crisis [9]. Despite the limitations of both studies, these results are worth taking into account when making the decision about the mechanical ventilatory therapy to employ in the myasthenic patient.

It must be taken into consideration that the main causes of death in myasthenic crisis are lethal arrhythmias and pulmonary embolism [3]. Our patient was maintained with continuous hemodynamic monitoring and remained under prophylactic anticoagulation scheme his entire hospitalization. Weaning from noninvasive mechanical ventilation was conducted favoring the daily clinical evaluation, with the support of scores of assessment designed for myasthenic patient, using mainly the myasthenia gravis composite scale [10].

## 4. Conclusions

Myasthenic crisis is a neurological emergency. The presence of antibodies against acetylcholine and anti-MuSK in the same individual is an unusual form of presentation of the disease and confers increased therapeutic difficulty. Commonly, the presence of anti-MuSK antibodies is associated with bulbar symptoms, which tend to cause problems in swallowing and difficulty in handling secretions. The exacerbation of these symptoms is a strong inconvenience for the use of noninvasive mechanical ventilation. Our patient, however, by being supported by an intense respiratory therapy, and thanks to the timely start of immunomodulatory therapy, responded favorably to the treatment with noninvasive ventilation. We expect that the way the respiratory support and the immunomodulatory treatment were implemented in this case could serve other physicians who face a similar clinical situation.
